# Medical diagnoses among infants at entry in out‐of‐home care: A Swedish population‐register study

**DOI:** 10.1002/hsr2.133

**Published:** 2019-07-18

**Authors:** Ulf Högberg, Roland Sennerstam, Knut Wester, Göran Högberg, Jacob Andersson, Ingemar Thiblin

**Affiliations:** ^1^ Department of Women's and Children's Health Uppsala University Uppsala Sweden; ^2^ Department of Oncology and Pathology, Cancer Center Karolinska University Hospital and Karolinska Institutet Stockholm Sweden; ^3^ Department of Clinical Medicine—K1 University of Bergen Bergen Norway; ^4^ Department of Neurosurgery Haukeland University Hospital Bergen Norway; ^5^ Formerly Department of Women's and Children's Health, Child and Adolescent Psychiatric Unit Karolinska Institutet Stockholm Sweden; ^6^ Forensic Medicine, Department of Surgical Sciences Uppsala University Uppsala Sweden

**Keywords:** evidence‐based practice, infant welfare, medical errors, out‐of‐home care, shaken baby syndrome

## Abstract

**Background and aims:**

Identification of child abuse involves a medical investigation and assessment of problems related to social environment and upbringing and might necessitate out‐of‐home care. The objective of this study was to analyse infants placed in out‐of‐home care in Sweden by incidence, medical diagnoses, and perinatal factors.

**Methods:**

This was a population‐based register study of infants born in Sweden 1997 to 2014. Data were retrieved from registers at the Swedish National Board of Health and Welfare and Statistics Sweden. Outcome measures were out‐of‐home care categories: (a) “Problems Related to Social Environment/Upbringing”, (b) “Abuse diagnoses without SDH (subdural haemorrhage), RH (retinal haemorrhage), rib fracture, or long bone fracture”, and (c) “SDH, RH, rib fracture, or long bone fracture.” As a reference population, we randomly selected infants without medical diagnoses born the same year.

**Results:**

Overall incidence of out‐of‐home care was 402 per 100 000. For subcategories (a), (b), and (c), the incidences were 14.8 (n = 273), 3.77 (n = 70), and 9.83 (n = 182) per 100 000, respectively. During the study period, the first remained unchanged; the latter two have been increasing. Compared with other reasons for out‐of‐home care, children in category (c), “SDH, RH, rib fracture, or long bone fracture”, had increased odds of being boys (adjusted odds ratio [aOR] 1.60; 95% confidence interval [CI], 1.08‐2.38) and decreased odds of having a mother being single (aOR 0.49; 95% CI, 0.32‐0.75) and a smoker (aOR 0.60; 95% CI, 0.37‐0.96). Compared with the reference population, children in this category were more often twin born (7.7% versus 2.8%), preterm (18.5% versus 5.5%), and small‐for‐gestational age (5.2% versus 2.1%).

**Conclusion:**

SDH, RH, rib fracture, or long bone fracture constitute a minor part of medical diagnoses for infants entered in out‐of‐home care, but have been increasing, both in numbers and proportion. Overdiagnosis of abuse might be a possible reason but cannot be ascertained by this study design.

## 1 INTRODUCTION

Child health and child protection policy in Sweden has a long history, from the building of Child Care Centres from the 1930s, the forming of the Child Accident Prevention Committee in the 1950s, and prohibiting corporal punishment and emotional humiliation in 1979 to instituting the Children's Ombudsman in 1993.^1^ In 2001, the governmental committee for the “Child Abuse—Prevention and Protection” inquiry recommended establishment in all municipalities of a multisectoral and multidisciplinary agency, Barnahus (Children's House), linking the services of the police, social services, public prosecutor, children's and youth psychiatry, paediatrics, and forensic medicine.^2^ Further, a section of the Child Maltreatment division of the Swedish Paediatric Society promoted the establishment of Child Protection Teams in paediatric university departments, starting in 2007.^3^ In addition, clinical guidelines on shaken baby syndrome/abusive head trauma (SBS/AHT) were adapted for use in some parts of Sweden the same year,^4^, ^5^ and child care centres recommended informing parents about the dangers of shaking a baby.^5^, ^6^

Swedish welfare law mandates any professional to report to social services any harmful domestic condition that may expose a child to risks. For child maltreatment welfare, interventions involve out‐of‐home care in foster families or residential care; such measures can be voluntary (Social Service Act) or compulsory (Compulsory Care Act).

The incidence of out‐of‐home care for infants in Sweden during the years 1998 to 2009 was 275.7 per 100 000, while incidences in England, the United States, and Manitoba (Canada) were much higher: 696.4, 631.4, and 2913.1, respectively.^1^ For preschool children (0‐6 y) born in Sweden between 1992 and 1996, increased odds of out‐of‐home care were associated with the mother giving birth in her teens, single, less educated, unemployed, and with psychosocial adversity, but not with being a second‐generation immigrant.^7^

For Swedish children aged 1 to 6 years during the 1990s and early 2000s, trends in maltreatment indicated a decrease in parental reports of severe child abuse, admissions for maltreatment or assault, violent deaths, or adolescents reporting severe beating by parents.^1^ Yet, until 2009, little change was noted in rates of infant (aged 0‐1 y) maltreatment, out‐of‐home care, or deaths.^1^ However, this trend was broken by a doubling of infant abuse diagnoses from the period 1997 to 2007 to 2008 to 2014.^8^

The following diagnoses, subdural haemorrhage (SDH), retinal haemorrhage (RH), skull fracture, rib fracture, classic metaphyseal lesions (CMLs), long bone shaft fracture, apnoea, and seizures, are claimed to be specific for the diagnosis of abuse.^9^, ^10^, ^11^, ^12^, ^13^, ^14^, ^15^ However, the scientific solidity of the SBS/AHT diagnosis has been questioned.^16^, ^17^, ^18^, ^19^, ^20^, ^21^ A systematic literature review of the Swedish Agency for Health Technology Assessment and Assessment of Social Service (SBU) concluded that there is limited scientific evidence to explain the triad *or* its components (subdural haematoma, RHs, and encephalopathy) by isolated shaking.^22^ Moreover, there is insufficient evidence on which to assess the diagnostic accuracy of the triad in identifying SBS/AHT, irrespective of presumed injury mechanisms.^22^ This systematic literature review has been criticized, commented on, and answered by the SBU expert group.^23^, ^24^, ^25^, ^26^, ^27^, ^28^, ^29^ We have provided evidence of perinatal risk factor profiles of infants with abuse diagnosis and SDH,^30^ rib, or long bone fractures,^31^ risk profiles that are similar to those having a medical cause of SDH or fractures.

To our knowledge, out‐of‐home care, specifically among infants aged 0 to 1 years, has not been studied in Sweden with respect to medical diagnoses. It might be hypothesized that the observed increase in the diagnosis of abuse is not real but due to an overdiagnosis of abuse (false positives).^8^, ^22^, ^23^ The objective of this study was to analyse the following epidemiological aspects for out‐of‐home care for infants:

- the incidence of entries in out‐of‐home care, overall and by medical diagnoses, prior to or at the time of out‐of‐home care;
- perinatal and parental factors associated with the infants' entry into out‐of‐home care by medical diagnoses.

## 2 METHODS

### 2.1 Selection and description of participants

This is a nationwide population‐based register study that includes Swedish infants born between 1997 and 2014 with follow‐up to 1 year of age, and their parents, utilizing the health registers at the Swedish National Board of Health and Welfare^32^ and Statistics Sweden. The National Patient Register (NPR) covers in‐patient care from 1997 to 2015 and specialized out‐patient care (2001‐2015). During the study period, the NPR applied the International Classification of Diseases (ICD‐10).

A flow chart of the study design is presented in Figure S1. Out of 1 855 267 children born, 395 812 had an entry in NPR. From those, a selection of 119 diagnoses was made (n = 182 974 children).^8^ For analysis of perinatal and parental factors, four controls were selected for each included infant; these were born the same year and had no diagnoses in NPR during the first year of life.^8^ Information from the *Swedish Medical Birth Register* (SMBR) (1997‐2014), *Register of Children and Young Persons Subjected to Child Welfare Measures*, still referred to as the Out‐of‐home Care Register (1997‐2015), and *Educational Register* (Statistics Sweden) was linked with each personal identity number.

Of the final sample, 1514 infants had an entry in the Out‐of‐home Care Register (Figure S1).

To calculate the overall incidence of entries into out‐of‐home care, the number of all infants that had an entry in the *Register of Children and Young Persons Subjected to Child Welfare Measures* was retrieved as aggregated data without personal identity number or linkage to other registers within this study design (Figure S1).

### 2.2 Outcome measures

Out‐of‐home care was defined as the first entry into the Out‐of‐home Care Register and coded as a voluntary entry according to the Social Service Act (chapter 6, §1) or compulsory entry according to the Compulsory Care of Young Persons Act (§2, 3, and 6).

### 2.3 Exposures

We selected a total of 51 diagnoses of abuse, adverse social and parental circumstances, and specific diagnoses that might be associated with infant abuse according to the literature (Table S1).^1^, ^8^, ^11^, ^30^, ^31^, ^33^ Only specific diagnoses of abuse, or adverse social and parental circumstances preceding the date of first entry in the *Out‐of‐home Care Register* or within 15 days after that date, were categorized as exposure variables. Diagnoses of SDH, RH, rib fracture, or long bone fracture were selected that are claimed to have the highest positive predicative value for abuse, PPV of 0.69 for SDH,^9^ 0.97 for severe RH,^9^ 0.67 to 1.0 for rib fracture,^10^, ^15^, ^34^ and 0.57 for long bone fracture.^11^ Those diagnoses were combined in different categories and finally as one category, “SDH, RH, rib fracture, or long bone fracture” (Table 1).

**Table 1:** Specified infant diagnoses (ICD‐10) before or at time of out‐of‐home care (±15 d) according to the Social Service Act or Compulsory Care of Young Persons Act by superficial body or head injury and fall accidents for children aged 0‐1 y of age born in Sweden 1997‐2014

Diagnosis	All (n = 782)	Superficial Body Injury (n = 15)	Superficial Head Injury (n = 52)	Fall Accidents^a^
	n (%)	n (%)	n (%)	n (%)
Problems Related to Social Environment/Upbringing^b^	273 (34.9)	2 (0.7)	5 (1.8)	17 (6.2)
Abuse diagnosis but neither subdural haemorrhage, retinal haemorrhage, rib fracture, long bone fracture	70 (9.0)	4 (5.7)	2 (2.9)	6 (8.6)
Assault^c^	13 (1.7)	2 (15.4)	1 (7.7)	0
Superficial injury^d^				
Superficial body injury or bruises	15 (1.9)	15 (100)	1 (6.7)	4 (27)
Burns	6 (0.8)	0	0	1 (17)
Head injuries, cranial, and CNS diagnoses^e^				
Superficial head injury	52 (6.7)	1 (1.9)	52 (100)	35 (67)
Subdural haemorrhage	63 (8.1)	0	2	16 (25)
Epidural haemorrhage^f^	6 (0.8)	0	0	2 (33)
Subarachnoidal haemorrhage^g^	9 (1.2)	0	0	2 (22)
Skull fracture	51 (6.5)	1 (2.0)	2 (3.9)	26 (51)
Cerebral contusion	29 (3.7)	0	2 (6.9)	24 (83)
Retinal haemorrhage	32 (4.1)	0	0	5 (16)
Acute life threatening events	33 (4.2)	1 (3.0)	0	1 (3)
Seizures	53 (6.8)	1 (1.9)	3 (5.7)	7 (13)
Fractures				
Long bone fracture^h^	81 (10.4)	2 (2.5)	1 (1.2)	29 (36)
Rib fracture	34 (4.4)	0	1	6 (18)
Clavicle fracture	16 (2.1)	1 (6.3)	0	5 (31)
Others				
Failure‐to‐thrive	70 (9.0)	0	1 (1.4)	4 (5.8)
Composite				
Subdural haemorrhage + retinal haemorrhage	23 (2.9)	0	0	4 (17)
Subdural haemorrhage + retinal haemorrhage + cerebral contusion	1 (0.4)	0	0	0
Subdural haemorrhage + skull fracture	12 (1.5)	0	1 (8.3)	4 (33)
Long bone fracture + rib fracture	19 (2.4)	0	0	6 (32)
Subdural haemorrhage + long bone fracture	8 (1.0)	0	0	2 (25)
Subdural haemorrhage + rib fracture	6 (0.8)	0	0	2 (33)
Subdural haemorrhage + long bone fracture + rib fracture	5 (0.6)	0	0	2 (40)
Any: Subdural haemorrhage, retinal haemorrhage, rib fracture, long bone fracture	182 (23.3)	2 (1.1)	6 (3.3)	67 (37)

Abbreviations: CNS, central nervous system; ICD, International Classification of Diseases.

a Fall accidents from same level, while being carried, chair, or other furniture, involving bed, involving stairs and steps and unspecified.

b Abuse diagnosis (12), had skull fracture (2), clavicle fracture (1).

c Maltreatment syndrome (4), skull fracture (1), subdural haemorrhage (1), cerebral contusion (1), long bone fracture (1), rib fracture (1).

d No case of black eye.

e No case of cervical fracture, sprain and strain cervical spine, injuries of brain and cervical nerves and spinal cord at neck level.

f Skull fracture (3), subdural haemorrhage (3), convulsions (1), none subarachnoidal haemorrhage.

g Skull fracture (2), subdural haemorrhage (3), convulsions (1).

h 40 were nonshaft long bone fractures.

Incidences of out‐of‐home care were estimated for all infants and following subcategories: (a) “Problems Related to Social Environment/Upbringing”, (b) “Abuse diagnoses without SDH, RH, rib fracture, or long bone fracture”, and (c) “SDH, RH, rib fracture, or long bone fracture”.

To analyse differences in perinatal and parental characteristics, the following categories of infants with entry into out‐of‐home care were selected: (1) infants with any medical diagnosis, (2) infants with “Problems Related to Social Environment/Upbringing”, and (3) infants with “SDH, RH, rib fracture, or long bone fracture”. These were compared with the reference population (see Table 2). To analyse risk factors, the categories “Problems Related to Social Environment/Upbringing” and “SDH, RH, rib fracture, or long bone fracture” were compared with out‐of‐home care children without those diagnoses (see Table 3).

**Table 2:** Infant, birth, and parental characteristics for infants born in Sweden 1997‐2014 with entry in out‐of‐home care by (a) diagnoses not related to “Problems Related to Social Environment/Upbringing” or subdural haemorrhage (SDH), retinal haemorrhage (RH), rib fracture, or long bone fracture, (b) diagnosis of “Problems Related to Social Environment/Upbringing,”^b^ and (c) “SDH, RH, rib fracture, or long bone fracture”^c^

Exposure		Reference Population (n = 730 971^a^)	Infant Entry in Out‐of‐Home Care (n = 1514)					
			(a) Diagnoses Not Related to Problems Related to Social Environment/Upbringing, or SDH, RH, Rib Fracture, or Long Bone Fracture (n = 1070)		(b) Problems Related to Social Environment/Upbringing^b^ (n = 262^d^)		(c) SDH, RH, Rib Fracture, or Long Bone Fracture^c^ (n = 169^e^)	
		n (%)	n (%)	P Value	n (%)	P Value	n (%)	P Value
	Female	356 811 (48.8)	496 (46.4)	0.011	128 (48.9)	0.989	63 (37.3)	0.003
	Male	374 154 (51.2)	574 (53.6)		134 (51.1)		106 (62.7)	
	Other	6	0		0		0	
Multiple birth		20 655 (2.8)	52 (4.9)	<0.001	12 (4.6)	0.087	13 (7.7)	0.001
	Missing	0	0		0		0	
Gestational week	>37	690 356 (94.5)	877 (82.6)	<0.001	208 (82.9)	<0.001	137 (81.5)	<0.001
	Preterm < 36	40 279 (5.5)	185 (17.4)		43 (17.1)		31 (18.5)	
	Missing	336	8		9		1	
SGA	<25th percentile	14 889 (2.1)	84 (7.9)	<0.001	17 (7.1)	<0.001	8 (5.2)	0.013
	Missing	22 275	66		24		19	
Parity	1	314 273 (43.0)	576 (53.8)	<0.001	128 (48.9)	0.002	93 (55.0)	0.001
	2‐3^f^	372 601 (51.0)	379 (35.4)^f^		101 (38.5)^f^		66 (39.1)^f^	
	>4^b^	44 092 (6.0)	115 (10.7)	<0.001	33 (12.6)		10 (5.9)	0.465
	Missing	5			0		0	
Age	<20	10 706 (1.5)	165 (15.4)	<0.001	26 (10.2)	<0.001	25 (14.8)	<0.001
	20‐34^f^	564 946 (77.3)	733 (68.5)^f^		166 (64.8)^f^		127 (75.1)^f^	
	>35	155 313 (21.2)	172 (16.1)	0.006	64 (25.0)	0.021	17 (10.1)^b^	0.004
	Missing	6	0		0		0	
BMI	Normal^f^	396 803 (60.6)	441 (50.6)^f^		83 (43.0)^f^		66 (44.6)^f^	
	Underweight (BMI < 19)	15 686 (2.4)	39 (4.5)	<0.001	8 (4.1)	0.013	7 (4.7)	0.009
	Overweight (BMI ≥ 25)	241 999 (37.1)	391 (44.9)	<0.001	102 (52.8)	<0.001	75 (50.7)	0.001
	Missing	76 483	199		69		21	
Smoking pregnancy week 30‐32	Nonsmoking	628 613 (94.6)	561 (67.9)	<0.001	114 (67.9)	<0.001	112 (81.8)	<0.001
	Smoking	35 578 (5.4)	265 (32.1)		54 (32.1)		25 (18.2)	
	Missing	66 780	244		94		32	
	Missing		124		20		30	
Family situation	Cohabitating	655 344 (94.2)	535 (56.3)	<0.001	107 (50.0)	<0.001	115 (72.8)	<0.001
	Single or other	40 450 (5.8)	416 (43.7)		107 (50.0)		43 (27.2)	
	Missing	35 177	119		48		11	
Mother country of birth	Nordic countries	621 817 (91.3)	658 (82.7)	<0.001	141 (84.9)	0.003	128 (88.3)	0.203
	Other	59 535 (8.7)	138 (17.3)		25 (15.1)		17 (11.7)	
	Missing	49 619	274		96		24	
Mother years in school	>14	277 133 (37.9)	71 (7.3)	<0.001	14 (5.8)	<0.001	23 (14.2)	<0.001
	10‐14	368 865 (52.0)	450 (46.2)		120 (49.6)		92 (56.8)	
	≤9	63 633 (8.7)	453 (46.5)		108 (44.6)		47 (29.0)	
	Missing	21 340	96		20		7	
Father years in school	>14	182 434 (25.0)	59 (6.7)	<0.001	9 (4.3)	<0.001	16 (10.2)	<0.001
	10‐14	436 132 (59.7)	483 (54.6)		118 (57.0)		92 (65.0)	
	≤9	79 465 (10.9)	342 (36.9)		80 (38.6)		39 (24.8)	
	Missing	32 940	186		55		22	

*Note.* *P* values are from Mantel‐Haenszel chi‐square test comparing all samples with reference population.^a^

Abbreviations: BMI, body mass index; RH, retinal haemorrhage; SDH, subdural haemorrhage; SGA, small‐for‐gestational age.

**Figure 1:**
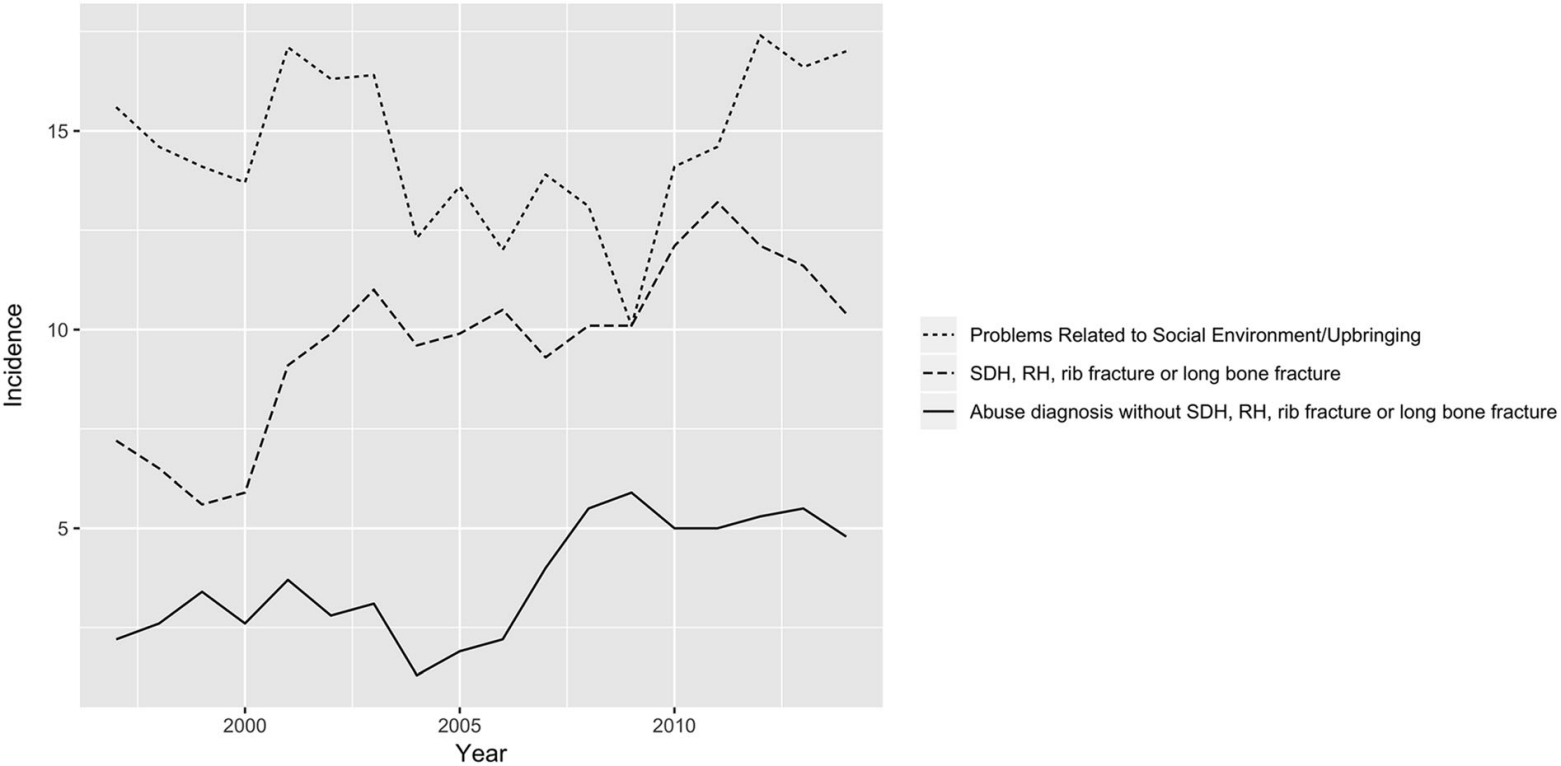
Trends in out‐of‐home care among infants born in Sweden 1997‐2014 per 100 000 infants (moving annual average) by diagnosis category: (1) “Problems Related to Social Environment/Upbringing” (problems related to social environment, negative life events in childhood, other problems related to upbringing, related to lifestyle, to care‐provider dependency, history of mental and behavioural disorders) (n = 273) (P value 0.97), (2) “Abuse diagnoses without SDH, RH, rib fracture, or long bone fracture” (n = 70) (P value 0.002), (3) “SDH, RH, rib fracture or long bone fracture” (n = 182) (P value < 0.001). P values are from chi‐square test for linear trend. RH, retinal haemorrhage; SDH, subdural haemorrhage

a Population information from infants having no entry in Patient Register during first year of life (Figure 1).

b Problems related to social environment, negative life events in childhood, other problems related to upbringing, related to lifestyle, to care‐provider dependency, persons encountering health services in other circumstances, family history of mental, and behavioural disorders.

c Subdural haemorrhage, retinal haemorrhage, rib fracture, or long bone fracture.

d 11 out of 273 excluded because concomitant diagnosis of subdural haemorrhage, retinal haemorrhage, rib fracture, or long bone fracture.

e 13 out of 182 excluded because of concomitant diagnosis of Problems Related to Social Environment and Upbringing or diagnosis of superficial injury, Problems not related to Environment/Upbringing or subdural haemorrhage, retinal haemorrhage, rib fracture, or long bone fracture.

f Reference group for chi‐square test.

**Table 3:** Maternal and infant risk factors of out‐of‐home care for infants aged 0‐1 y born in Sweden 1997‐2014 by diagnosis category (1) “Problems Related to Social Environment/Upbringing” and (2) “Subdural haemorrhage (SDH), retinal haemorrhage (RH), rib fracture, or long bone fracture” with reference category not belonging to those categories^a^

Exposure		Problems Related to Social Environment/Upbringing^b^		SDH, RH, Rib or Long Bone Fracture^c^	
		n = 262		n = 169	
		OR (95% CI)	aOR^d^ (95% CI)	OR (95% CI)	aOR^d^ (95% CI)
Infant					
	Female	1		1	
	Male	0.91 (0.69‐1.19)	0.93 (0.66‐1.33)	1.45 (1.04‐2.03)	1.60 (1.08‐2.38)
	Single birth	1		1	
	Multiple birth	0.94 (0.49‐1.79)	1.08 (0.47‐2.49)	1.63 (0.87‐3.07)	1.84 (0.89‐3.80)
Mother					
	Cohabiting	1		1	
	Single or other	1.29 (0.96‐1.73)	1.44 (1.00‐2.06)	0.48 (0.33‐0.70)	0.49 (0.32‐0.75)
	Nonsmoker	1		1	
	Smoker	1.03 (0.70‐1.43)		0.47 (0.30‐0.75)	0.60 (0.37‐0.96)
	>9 y in school	1		1	
	≤9 y in school	0.93 (0.70‐1.23)	1.24 (0.61‐2.50)	0.47 (0.33‐0.68)	0.63 (0.36‐1.10)

*Note.* Crude (OR) and adjusted (aOR) odds ratios and 95% confidence intervals (CIs).

a Infant with entry in out‐of‐home with any medical diagnosis during infancy but not diagnoses of Problems Related to Social Environment/Upbringing and neither any subdural haemorrhage, retinal haemorrhage, long bone fracture, rib fracture (n = 1070).

b 11 out of 273 not included because concomitant diagnoses of subdural haemorrhage, retinal haemorrhage, long bone fracture, or rib fracture.

c 13 out of 182 not included because of concomitant diagnosis of Problems Related to Social Environment/Upbringing or diagnosis of superficial injury.

d Adjusted for sex, multiple birth, family situation, maternal smoking, and mother's years in school.

We defined the following perinatal (for the index pregnancy and birth) and parental variables according to current knowledge:^7^, ^8^, ^30^, ^31^

- Maternal and perinatal information: sex of infant; single/multiple birth, term, or preterm born (<37 gestational week); small‐for‐gestational age (SGA) (<2.5 percentiles); maternal body mass index (BMI) at start of index pregnancy, defining underweight (BMI < 19) or overweight (BMI ≥ 25); and maternal smoking in pregnancy weeks 30 to 32.
- Sociodemographic information: mother married/cohabiting or single living; maternal age <20, 20 to 34, >35 years; parity 1 and parity 2+; maternal birth country, either the Nordic countries (Denmark, Finland, Iceland, Norway, or Sweden) or outside these countries; and highest level of education for mother and father at time of birth of the index child as basic, secondary, or postsecondary education.

### 2.4 Statistics

Incidence proportion was calculated as cases per 100 000 infants. For time trends, moving annual averages were estimated. Chi‐square test was used to evaluate linear trends (extended Mantel‐Haenszel). Median lengths of out‐of‐home care are presented with descriptive statistics. Missing data were entered as a separate category within each variable. Mantel‐Haenszel chi‐square test, two‐sided, was applied to assess differences between exposures and outcomes for each of the three categories. Alpha level for statistical significance was 0.05. Logistic regressions were used for risk factor analyses: We present both crude odds ratios and odds ratios adjusted according to current knowledge,^6^, ^7^, ^18^, ^19^ with 95% confidence intervals (CIs).

The statistical software packages R version 1.2.1114 (figures) and IBM SPSS 25·0 (chi‐square and regression) (SPSS Inc, Armonk, New York, IBM Corp) were used for statistical analyses.

### 2.5 Ethics

The Regional Ethical Committee in Uppsala approved the study (2014‐11‐19 no. 383). Register linkage was provided by the National Board of Health and Welfare. This committee approved a waiver of informed consent, considering that the research database contained only coded data.

## 3 RESULTS

During the study period, a total of 7455 infants born in Sweden between 1997 and 2014 were enrolled in out‐of‐home care during their first year of life. Of those, 77.8% had interventions according to the Social Service Act and 22.2% according to the Compulsory Care Act. The incidence proportion of out‐of‐home care of infants during the study period was 402 per 100 000, with a statistically significant increase from 328 in 1997 to 439 per 100 000 infants in 2014 (*P* < 0.001, chi‐square for trend).

### 3.1 Out‐of‐home care by medical diagnoses

In our sample, 782 of all infants in out‐of‐home care (51.6%) had any of the 51 prespecified diagnoses (see Section 2—Figure S1 and Table S1). The most common medical diagnoses were head injury and craniocerebral conditions (41.9%, 328/782) followed by fractures (16.9%, 131/782) and failure‐to‐thrive (9.0%, 9/782). Only few children in our sample of 782 had diagnoses related to superficial injuries of the body (1.8%) or burns (0.8%) (Table 1).

The most common category was “Problems Related to Social Environment/Upbringing” (34.9%, 273/782), followed by “SDH, RH, rib fracture, or long bone fracture” (23.3%, 182/782), and “Abuse diagnosis without any diagnoses of SDH, RH, rib fracture, or long bone fracture” (9.0%, 70/782); for single, isolated diagnoses, the frequencies were long bone fracture (10.4%), SDH (8.1%), skull fracture (6.5%), and RH (4.1%). By combination of diagnoses, there were few children having both SDH and RH (2.9%) and only one had SDH, RH, and cerebral contusion (0.4%).

A high proportion of infants with the diagnoses of head injuries, cranial, or CNS diagnoses had reported fall accidents: SDH (25%), skull fracture (51%), and cerebral contusion (83%). A high proportion of fracture diagnoses had reported fall accidents: long bone fracture (36%), rib fracture (18%), and clavicle fracture (31%). In the category “SDH, RH, rib fracture, or long bone fracture,” 37% had a reported fall accident. Few of the categories had a specific diagnosis of a superficial injury; this was most frequently recorded for those in the “Abuse diagnoses without diagnoses of SDH, RH, rib fracture, or long bone fracture” category (8.6%). Infants with “SDH, RH, rib fracture, or long bone fracture” were more often (47.3%, 86/182) enrolled in out‐of‐home care under the Compulsory Care Act than infants with “Problems Related to Social Environment/Upbringing” (28.6%, 78/273) (*P* < 0.001, chi‐square).

Median ages at entry into out‐of‐home care for infants in “Problems Related to Social Environment/Upbringing”, “Abuse diagnoses without SDH, RH, rib fracture, or long bone fracture”, or “SDH, RH, rib fracture, or long bone fracture” were 1, 3.3, and 4 months, respectively.

### 3.2 Incidence and time trend by diagnosis category

The incidences during the study period for the out‐of‐home care categories “Problems Related to Social Environment/Upbringing,” “Abuse diagnoses without SDH, RH, rib fracture, or long bone fracture,” and “SDH, RH, rib fracture, or long bone fracture” were 14.8, 3.77, and 9.83 per 100 000, respectively. The annual incidence for “Problems Related to Social Environment/Upbringing” remained stable during the study period, whereas it increased for “Abuse diagnoses without SDH, RH, rib fracture, or long bone fracture” (*P* value 0.002) and “SDH, RH, rib fracture, or long bone fracture” (*P* value < 0.001) (chi‐square for trend); see Figure 1.

### 3.3 Background factors associated with out‐of‐home care

Table 2 shows the distribution of perinatal and parental factors in our out‐of‐home care samples by the categories “Problems Related to Social Environment/Upbringing” and “SDH, RH, rib fracture, or long bone fracture”, and an additional category, with children with none of those. Compared with the reference population, children in out‐of‐home care had a statistically significant higher proportion of boys, being multiple born (4.9%‐7.7% versus 2.8%), preterm (17.1%‐18.5% versus 5.5%), and SGA (5.2%‐7.9% versus 2.1%). Their mothers were more often primipara or multipara (4+ children), young, underweight or overweight, smokers (18.2%‐32.1% versus 5.4%), single (27.2%‐50% versus 5.8%), not Nordic‐born (11.7%‐17.3% versus 8.7%), and less educated (≤9 y in school) (29.0%‐46.5% versus 8.7%); the latter was also for their fathers (24.8%‐38.6% versus 10.9%).

Table 2 shows that infants within the category “SDH, RH, rib fracture, or long bone fracture” had a marked male preponderance (62.7%) and multiple births (7.7%); their mothers were less often smokers, more often living together, and had attended school for more years than other mothers with infants in out‐of‐home care.

Compared with other infants in out‐of‐home care, infants in the “SDH, RH, rib fracture, or long bone fracture” category had increased odds of being boys (adjusted odds ratio [aOR] 1.60; 95% CI, 1.08‐2.38) and had decreased odds of having a mother who smoked (aOR 0.60; 95% CI, 0.37‐0.96) and was single (aOR 0.49; 95% CI, 0.32‐0.75) (Table 3).

## 4 DISCUSSION

The overall incidence of out‐of‐home care for infants was 402 per 100 000 during the study period (1997‐2014). For the category “Problems Related to Social Environment/Upbringing”, the incidence was 14.8 per 100 000, with no increase during this period; for categories “Abuse diagnosis without subdural haemorrhage, retinal haemorrhage, rib fracture, or long bone fracture” and “SDH, RH, rib fracture, or long bone fracture”, the incidences were 3.77 and 9.83 per 100 000, respectively, and both increased during the study period. In the category “SDH, RH, rib fracture, or long bone fracture”, 37% of the infants had a reported fall accident. Parents of infants in the total out‐of‐home care sample had a typically adverse perinatal and socioeconomic profile compared with the population, while parents to infants in the “SDH, RH, rib fracture, or long bone fracture” group were better educated, more often living together, and the mothers smoked less than mothers of other infants in out‐of‐home care.

The overall incidence of all infants with first entry into out‐of‐home care, although increasing during the study period, was comparable with that in Western Australia 1994 to 2005,^35^ slightly lower than that in England 1995 to 2008, and higher than that in Denmark, where the incidence has been declining.^36^

This is the first Swedish study addressing medical diagnoses and out‐of‐home care among infants. Medical diagnoses were infrequent among the total number of infants entered; 2.4% had diagnoses compatible with SBS/AHT criteria as SDH, RH, rib fracture, or long bone fracture, and 0.9% had an abuse diagnosis without any of those SBS/AHT criteria, similar to the proportions reported from Western Australia^31^ and Manitoba.^1^

The high proportion (37%) of fall accidents among those having SDH, RH, rib fracture, or long bone fracture is intriguing. Swedish population data show that fall accidents, mostly with slight or moderate trauma, are reported for 34% of all SDH diagnoses and for 71% of all long bone fractures.^30^, ^31^ However, the diagnostic considerations of abuse while parents have reported a fall accident cannot be addressed by this study design. According to the Swedish SBS/AHT guidelines from 2008, the triad is caused by abuse provided traffic accident and fall from high altitude can be excluded.^5^

A maternal socioeconomic risk profile was evident for infants in the “Problems Related to Social Environment/Upbringing” sample, as previously reported for out‐of‐home care.^7^, ^35^, ^37^ This pattern was, however, less pronounced for infants associated with SBS/AHT criteria, who, surprisingly, had parents who scored better for education, living together, and smoking. Biological risk factors such as smoking,^38^ SGA,^39^ obesity,^40^ and prematurity^39^ are known to be more prevalent among socioeconomically disadvantaged individuals, and socioeconomic factors are also associated with child morbidity^41^ and child mortality.^42^ SGA and prematurity are associated with SDH,^30^ and in addition to these, obesity is associated with infant metabolic bone disease.^31^ Thus, biological risk factors might, at least to some extent, account for the relative overrepresentation of low socioeconomic status in the SBS/AHT criteria sample, compared with the reference population. There is also a possibility of selection bias related to doctors' inclination to interpret findings as being related to physical abuse among socially underprivileged carers.^43^

Our finding of an increase over time in out‐of‐home care associated with SBS/AHT criteria is intriguing in view of the fact that information about the dangers of shaking was introduced to parents during the study period^6^ and parental reports of shaking decreased from 18% in 2006 to 0% in 2011.^8^

The risk factors for infants in out‐of‐home care in association with SDH, RH, rib fracture, or long bone fracture have similarities with previously reported risk factors for SBS/AHT, such as preterm,^8^, ^33^ male preponderance,^9^ and multiple birth.^8^, ^33^, ^44^ This might be interpreted that having a boy, caring of a preterm, or having twins are potential predictors of provoking violence. An alternative explanation is that these characteristics are associated with medical conditions that predispose to the spontaneous occurrence of physical findings that are also included in the SBS/AHT criteria. Given that only a small proportion (1.8%) of the infants had superficial injuries of the body indicating violence, it is possible that a considerable proportion of those infants had such underlying medical conditions. This assumption is further supported by the fact that diagnoses of SDH, long bone, and rib fracture that were associated with abuse only constituted a minor part of all those fractures found in the population, as shown in our previous studies.^30^, ^31^

Only one case had the triad (SDH, RH, and encephalopathy), and rather few had a combination of diagnoses. The number of infants with “SDH, RH, rib fracture, or long bone fracture” in this study might be interpreted as correctly indicating infant abuse and proper out‐of‐home care intervention, provided that the prevailing SBS/AHT paradigm employs evidence‐based practice.^3^, ^9^, ^10^, ^11^, ^12^, ^13^, ^33^ However, the scientific solidity of the SBS/AHT paradigm has been challenged.^17^, ^18^, ^19^, ^20^, ^21^, ^22^, ^45^ Further, the claimed high predictivity of long bone and rib fractures for diagnosing SBS/AHT^11^, ^12^ has been challenged by previous reviews and described “to be of low quality (high risk of bias)” because of circular reasoning.^22^

If the parents cannot provide a plausible trauma history that explains the medical findings, this is believed^33^, ^46^—but not scientifically verified^22^—to signify physical abuse, thus implying that the reason for out‐of‐home care could have been based on overdiagnosis of abuse in a substantial number of cases.

### 4.1 Implications

Correct diagnosis of infant abuse comprises the ethical principles of beneficence, nonmaleficence, and justice. Geographical differences in proportion of abuse diagnosis for SDH, rib fractures, and long bone fractures may be due to differences in diagnostic practices and do raise the possibility of overdiagnosis.^47^, ^48^, ^49^ If wrongful diagnostics and interpretations have been the reason for a decision to refer to out‐of‐home care, the implications for a family that only had been seeking health care for their infant are disastrous. This study gives evidence that diagnoses within the SBS/AHT paradigm constitute a considerable part of the diagnoses associated with that part of out‐of‐home care that is based on medical diagnoses. With respect to evidence‐based practice, it is conceivable that the current child protection policy in health care and the decisions made by the social welfare and judiciary systems might have led to infants being enrolled in out‐of‐home care that were wrongfully classified as being abused. This risk calls for further investigations including judiciary, medical, and social science competencies, examining each incident individually where AHT criteria have been the reason for referral to out‐of‐home care.

### 4.2 Strengths and weaknesses of the study

The strength of this study is the population design: The diagnoses were retrieved nationally, on the basis of a uniform ICD‐10 version. The reference population was representative, containing 39.4% of all children born during the study period. The reliability of the data drawn from the out‐of‐home care entries in the register has previously been reported to be satisfactory.^7^ The validity of the Swedish health registers is considered to be high, both with respect to the SBMR^50^ and the NPR.^51^ However, the specific diagnoses in this study have not yet been validated, and there are probably hidden cases of bruises, for example. The ICD‐10 does not differentiate CMLs from other long bone fractures; this is also a limitation of this study.

There are several other limitations and uncertainties in this study that deserve attention. The Swedish Out‐of‐home Care Register does not contain the actual causes for removing infants from their families; thus, our derivation of specific medical diagnoses from the NPR may not reflect the actual cause for out‐of‐home care. However, the applied diagnoses SDH, RH, rib fracture, or long bone fracture were recorded in accordance with stated knowledge,^9^, ^10^, ^11^, ^30^ thus supporting a causal inference.^8^

A major limitation is the lack of access to clinical records for assessment of medical diagnoses. When comparing the present study with our previous studies on SDH^26^ and rib and long bone fractures,^27^ there appears to be a certain degree of underreporting of abuse diagnoses categories in previous studies that were based on NPR: 63 versus 43 SDH, 81 versus 58 long bone fractures, and 34 versus 28 rib fractures. Whether an abuse diagnosis was made but not registered, or whether factors not shown in the registers have indicated abuse in infants with SBS/AHT criteria, remains uncertain.

For maternal background, only information about the index infant and parental education was used. Our analyses of socioeconomic variables were limited to educational background and household status; we had no access to the Total Income Enumeration Register.

## 5 CONCLUSIONS

Diagnoses of SDH, RH, rib fracture, or long bone fracture constitute a minor part of the overall sample of infants in out‐of‐home care but have increased considerably over the recent years. Overdiagnosis of abuse might be possible but cannot be ascertained by this study design. Overdiagnosis of abuse is not according to the ethical principles of beneficence, nonmaleficence, and justice.

## FUNDING

This study was supported by a grant from the Grieg Foundation, Bergen, Norway, to K.W. The funding source had no involvement in the research process of this study. The funding source had no involvement in the study design; collection, analysis, and interpretation of data; writing of the report; or the decision to submit the report for publication.

## CONFLICTS OF INTEREST

None declared.

## AUTHOR CONTRIBUTIONS

Conceptualization: Ulf Högberg, Roland Sennerstam, Göran Högberg, Jacob Andersson, Knut Wester, Ingemar Thiblin

Data curation: Ulf Högberg

Formal analysis: Ulf Högberg

Writing – original draft preparation: Ulf Högberg

Writing – review and editing: Ulf Högberg, Knut Wester, Roland Sennerstam,

Göran Högberg, Jacob Andersson, Ingemar Thiblin

All authors have read and approved the final version of the manuscript.

Ulf Högberg had full access to all of the data in this study and takes complete responsibility for the integrity of the data and the accuracy of the data analysis

## TRANSPARENCY STATEMENT

Ulf Högberg affirms that this manuscript is an honest, accurate, and transparent account of the study being reported; that no important aspects of the study have been omitted; and that any discrepancies from the study as planned (and, if relevant, registered) have been explained.

## DATA AVAILABILITY

The register data used in this study cannot be made publicly available directly by the authors. The data for this research project was obtained from the National Board of Health and Welfare in Sweden, which does not permit data sharing by the authors, according to the Swedish Secrecy Act 24:18. Interested researchers may request to access this data by directly contacting the National Board of Health and Welfare (socialstyrelsen@socialstyrelsen.se)

## Supporting information

Fig S1. Flow chart of the study base. Source: 1) Swedish National Board of Health and Welfare: National Patient Register (NPR), Medical Birth Register (MBR), Register of Children and Young Persons Subjected to Child Welfare Measures, 2) Statistics Sweden: Educational Register.

Table S1. Diagnoses according to International Classification of Diseases, version 10 (ICD10), National Patient Register, Swedish National Board of Health and Welfare.
