# Supplementary figures and images for: Medical diagnoses among infants at entry in out‐of‐home care: A Swedish population‐register study
Source: Health Sci Rep. 2019 Jul 18;2(8):e133. doi: 10.1002/hsr2.133 (PMC6707026; doi:10.1002/hsr2.133)

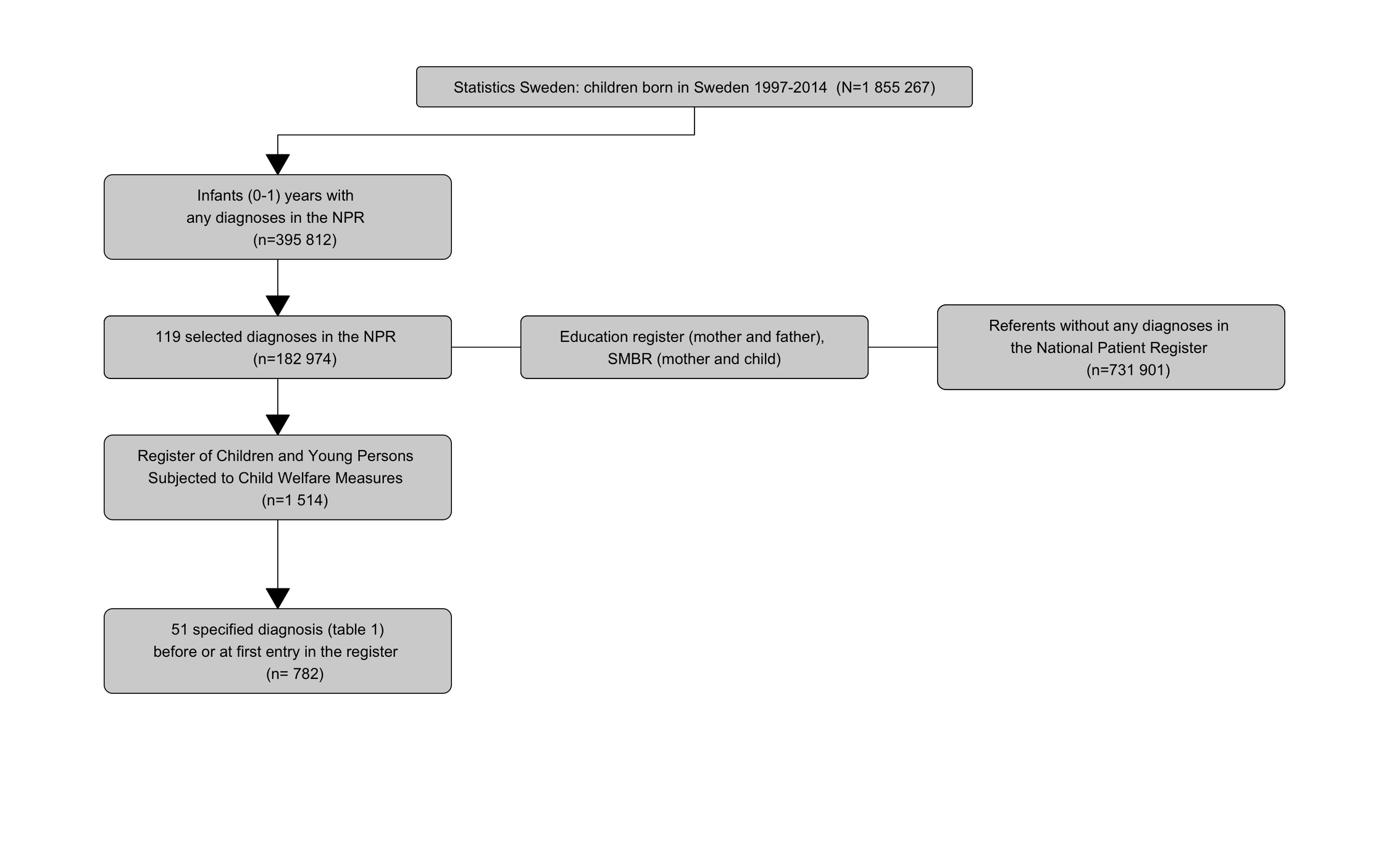

Supplement: Supplementary file 1 — Fig S1. Flow chart of the study base. Source: 1) Swedish National Board of Health and Welfare: National Patient Register (NPR), Medical Birth Register (MBR), Register of Children and Young Persons Subjected to Child Welfare Measures, 2) Statistics Sweden: Educational Register. [file HSR2-2-e133-s001.tif]
