# Supplementary material for: Medical diagnoses among infants at entry in out‐of‐home care: A Swedish population‐register study
Source: Health Sci Rep. 2019 Jul 18;2(8):e133. doi: 10.1002/hsr2.133 (PMC6707026; doi:10.1002/hsr2.133)
Supplement: Supplementary file 2 — Table S1. Diagnoses according to International Classification of Diseases, version 10 (ICD10), National Patient Register, Swedish National Board of Health and Welfare. [file HSR2-2-e133-s002.docx]

**Table S1.** Diagnoses according to International Classification of Diseases, version 10 (ICD10), National Patient Register, Swedish National Board of Health and Welfare.

|  | **Diagnosis category** | **ICD10** |
| --- | --- | --- |
| Infant abuse diagnoses & Problems Related to Social Environment/ Upbringing |  |  |
|  | Infant abuse diagnoses (observation for suspected abuse, battered baby syndrome, maltreatment syndrome) | Z03.8K, Y07, T74.1, Y06, Y07 |
|  | Problems Related to Social Environment/Upbringing (Problems related to social environment, negative life events in childhood, other problems related to upbringing, related to lifestyle, to care-provider dependency, person encountering health services in other circumstances, family history of mental and behavioral disorders) | Z60-Z62, Z72, Z74, Z76, Z81 |
|  |  |  |
| Superficial injuries |  |  |
|  | Superficial injury of unspecified body region | T14.0, T14A |
|  | Black eye | S001 |
|  | Burns | T20-32 |
| Head injuries, cranial and CNS diagnoses |  |  |
|  | Superficial head injury | S009, S208 |
|  | Retinal haemorrhage (RH) | H35.6 |
|  | Subdural haemorrhage (SDH) | I62.0 I62.9, S065, S065.0 |
|  | Epidural haemorrhage | S06.4, I62.1 |
|  | Subarachnoid haemorrhage | S06.6, I60 |
|  | Cervical fracture, sprain and strain cervical spine, injuries of brain and cervical nerves and spinal cord at neck level | S12, S13, T06.0 |
|  | Skull fracture | S020, S021, S028, S0209 S0200, S029 |
|  | Cerebral contusion | S060, S061 |
|  | Apparent life-threatening event in infant (ALTE) | P28.4, J96, R09.2 |
|  | Convulsions | R56, R56.8, G40–41, R56.0 |
| Fractures |  |  |
|  | Long bone fracture | S42.2, S42.3, S42,4, S42.7, S42.8, S52, S72, S82 |
|  | Rib fracture | S22.3, S22.4 |
| Others |  |  |
|  | Failure to thrive | R62.8 |
|  |  |  |
